# Gene regulatory pattern analysis reveals essential role of core transcriptional factors’ activation in triple-negative breast cancer

**DOI:** 10.18632/oncotarget.15749

**Published:** 2017-02-27

**Authors:** Li Min, Cheng Zhang, Like Qu, Jialiang Huang, Lan Jiang, Jiafei Liu, Luca Pinello, Guo-Cheng Yuan, Chengchao Shou

**Affiliations:** ^1^ Key Laboratory of Carcinogenesis and Translational Research (Ministry of Education), Departments of Biochemistry and Molecular Biology, Peking University Cancer Hospital & Institute, Beijing 100036, P. R. China; ^2^ Department of Biostatistics and Computational Biology, Dana-Farber Cancer Institute, Boston, MA 02115, USA; ^3^ Harvard T. H. Chan School of Public Heath, Boston, MA 02115, USA

**Keywords:** gene regulatory pattern, network analysis, transcriptional factors, TNBC

## Abstract

**Background:**

Triple-negative breast cancer (TNBC) is an aggressive breast cancer subtype. Genome-scale molecular characteristics and regulatory mechanisms that distinguish TNBC from other subtypes remain incompletely characterized.

**Results:**

By combining gene expression analysis and PANDA network, we defined three different TF regulatory patterns. A core TNBC-Specific TF Activation Driven Pattern (TNBCac) was specifically identified in TNBC by computational analysis. The essentialness of core TFs (ZEB1, MZF1, SOX10) in TNBC was highlighted and validated by cell proliferation analysis. Furthermore, 13 out of 35 co-targeted genes were also validated to be targeted by ZEB1, MZF1 and SOX10 in TNBC cell lines by real-time quantitative PCR. In three breast cancer cohorts, non-TNBC patients could be stratified into two subgroups by the 35 co-targeted genes along with 5 TFs, and the subgroup that more resembled TNBC had a worse prognosis.

**Methods:**

We constructed gene regulatory networks in breast cancer by Passing Attributes between Networks for Data Assimilation (PANDA). Co-regulatory modules were specifically identified in TNBC by computational analysis, while the essentialness of core translational factors (TF) in TNBC was highlighted and validated by *in vitro* experiments. Prognostic effects of different factors were measured by Log-rank test and displayed by Kaplan-Meier plots.

**Conclusions:**

We identified a core co-regulatory module specifically existing in TNBC, which enabled subtype re-classification and provided a biologically feasible view of breast cancer.

## INTRODUCTION

Breast cancer subtyping was widely used in clinical decisions, such as relapse risk evaluation and treatment selection [1, 2]. According to the evaluation of estrogen receptor (ER), progesterone receptor (PR) and human epidermal growth factor receptor 2 (HER-2/ERBB2/Neu), breast cancers are routinely divided into hormone receptor positive, HER-2/Neu amplified, and triple-negative breast cancer (TNBC) subtypes [2–4]. TNBC is particularly aggressive, thus often associated with relapse and the worst prognosis [3]. Due to a lack of appropriate molecular targets, TNBC patients could not benefit from endocrine or HER2-targeted therapy [5–7].

Multiple molecular characteristics of TNBC have been well identified [8–12], however, most studies were conducted from the perspective of gene expression, which cannot reflect the whole scope of pathologic mechanisms on gene regulation level, consequently, many questions of TNBC remain unanswered [13]. Recent systemic-level network analyses have been applied for diseases study and provide significant insights [14–16]. By incorporating multiple sources of data to model biological processes, especially transcriptional factor (TF) -gene regulatory networks, integrative analyses show promising perspective in comprehending of pathophysiologic mechanisms and developing novel and precise therapies [16, 17]. Among the multiple integration tools, Passing Attributes between Networks for Data Assimilation (PANDA) has better performance and higher accuracy [18–22]. PANDA predicts TF-gene regulatory relationships by integrating information from protein-protein interaction (PPI), gene expression, and TF-sequence-motif data using a message-passing approach, and it has been successfully used to study several diseases including Chronic Obstructive Pulmonary Disease (COPD) [23] and ovarian cancer [24].

In this study, we applied PANDA to characterize the gene regulatory network underlying TNBC, integrating datasets from The Cancer Genome Atlas (TCGA) database [25, 26]. In addition, we validated our predictions by using independent datasets obtained from Cancer Cell Line Encyclopedia (CCLE) [27, 28], Achilles [29, 30], Gene Expression Omnibus (GEO) [31] and Netherlands Cancer Institute (NKI) [32]. Our network approach identified a previously unrecognized core module containing 5 TFs and 35 target genes, thereby providing new mechanistic insights into TNBC. These insights are useful for prognosis as well as development of new therapeutic methods.

## RESULTS

### Building TF-target regulatory networks of NORM, nTNBC and TNBC

**Figure 1:**
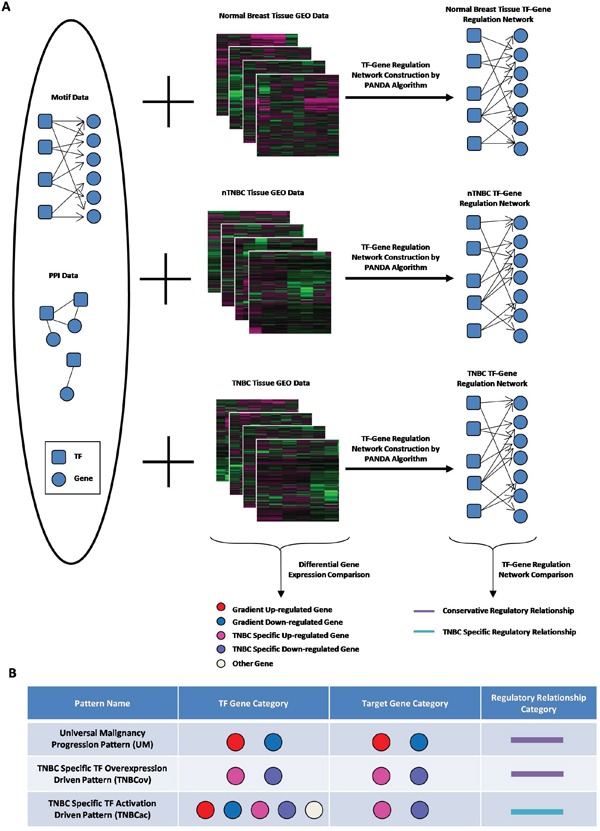
Outline of pattern finding approach **A.** Conceptual illustration summary of network construction and data processing; **B.** Cartoon chart to exhibit different regulation pattern we defined

Expression data for 63 NORM, 445 nTNBC and 89 TNBC tissue samples were extracted from TCGA. Robust multichip average (RMA) method [12, 33] was used for normalization and all probes were mapped to Ensembl Gene Symbols by R package mygene. Separate TF-target regulatory networks for the three tissue types were constructed by PANDA. An overview of the analysis pipeline is shown in Figure 1.

**Figure 2:**
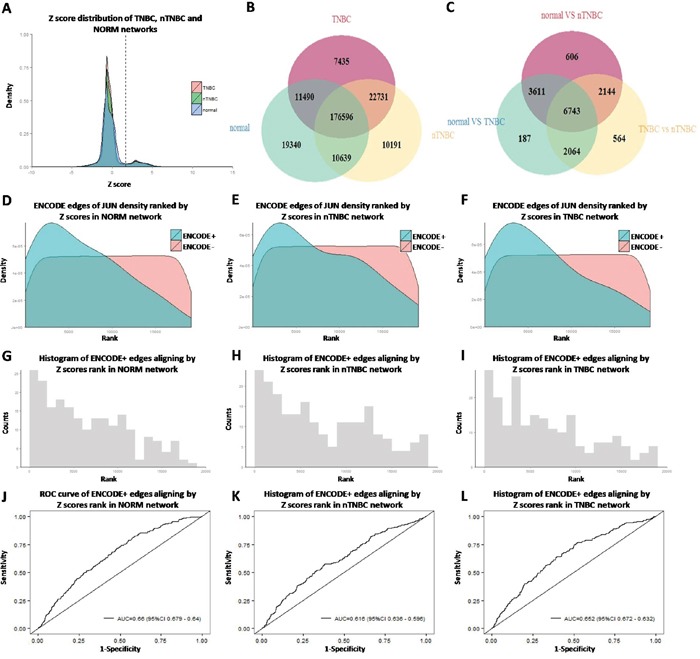
Gene regulatory network construction and validation **A.** Edge Z score distribution of different group; **B.** The overlap of edges between different groups; **C.** The overlap of differential expressed genes between different comparison; **D.E.F.** Density distribution of edges aligning by Z score rank, grouped by ENCODE ChIP-seq data (normal, nTNBC, TNBC); **G.H.I.** Histogram of ENCODE edges aligning by Z score rank of PANDA network (normal, nTNBC, TNBC); **J.K.L.** ROC curve of ENCODE edges aligning by Z score rank of PANDA network (normal, nTNBC, TNBC)

For each TF-target edge, a Z-score was given to reflect the confidence level of the potential regulatory relationship. Distribution of Z-scores in different groups was shown in Figure 2A. All edges with an FDR-adjusted *p*<0.05 were considered significant and used for the subsequent analysis. The overlap of significant edges between the tissue-specific networks was displayed as a Venn diagram (Figure 2B). More than 80% of TF-target edges were commonly shared among all three networks, indicating strong conservation, much higher compared to the overlap of differently expressed genes (Figure 2C).

Furthermore, ENCODE data were downloaded to validate the TF-target edges identified from our computational analysis. Since only two breast cancer cell lines were available in the ChIP-seq database, we chose to verify common edges in all cancer cells rather than in breast cancer cells only. For each TF, its target genes in each cell line were determined as those containing at least one peak in its promoter region (defined as [−750,+250] base-pairs around the transcription start site of an Ensembl Gene). Genes targeted in more than five cell lines were considered as common targets. We then compared the overlap between the ChIPseq-defined target genes and those predicted by PANDA. Take JUN, an evolutionarily conservative TF as an example, most of its common targets were ranked among the top 20% in our PANDA predicted networks (Figure 2D–2L, AUC>0.6), indicating our predictions were reasonable although not completely accurate. The complete results for validation were shown in Supplementary Figure 1.

### Identification and TFs co-regulation analysis of three distinct patterns

All genes’ expression profiles were pairwisely compared among NORM, nTNBC and TNBC by t-test, while genes with FDR<0.1 were considered differentially expressed. By combining the differential expression data and three networks together, three regulatory patterns were identified (Figure 1B): First, the Universal Malignancy Progression Pattern (UM) was defined as general biological processes during tumor progression, for which both TF and its targets were stepwise up/down-regulated from NORM to nTNBC to TNBC, in accordance with tissue malignancy change. These links are shared in all three tissue types (Figure 1B, first line). Second, the TF Overexpression Driven TNBC-Specific Pattern (TNBCov) was defined as those edges for which both the TF and its targets were up/down-regulated only in TNBC tissues (Figure 1B, second line). This pattern is associated with the effect of TF over-expression. Third, the TF Activation Driven TNBC-Specific Pattern (TNBCac) was defined as those edges for which the TF-target links were present only in the TNBC networks and the target genes were differentially expressed only in TNBC tissues (Figure 1B, third line). This pattern mimics a driving process in TNBC caused by TNBC specific TF activation or other functional changes.

**Figure 3:**
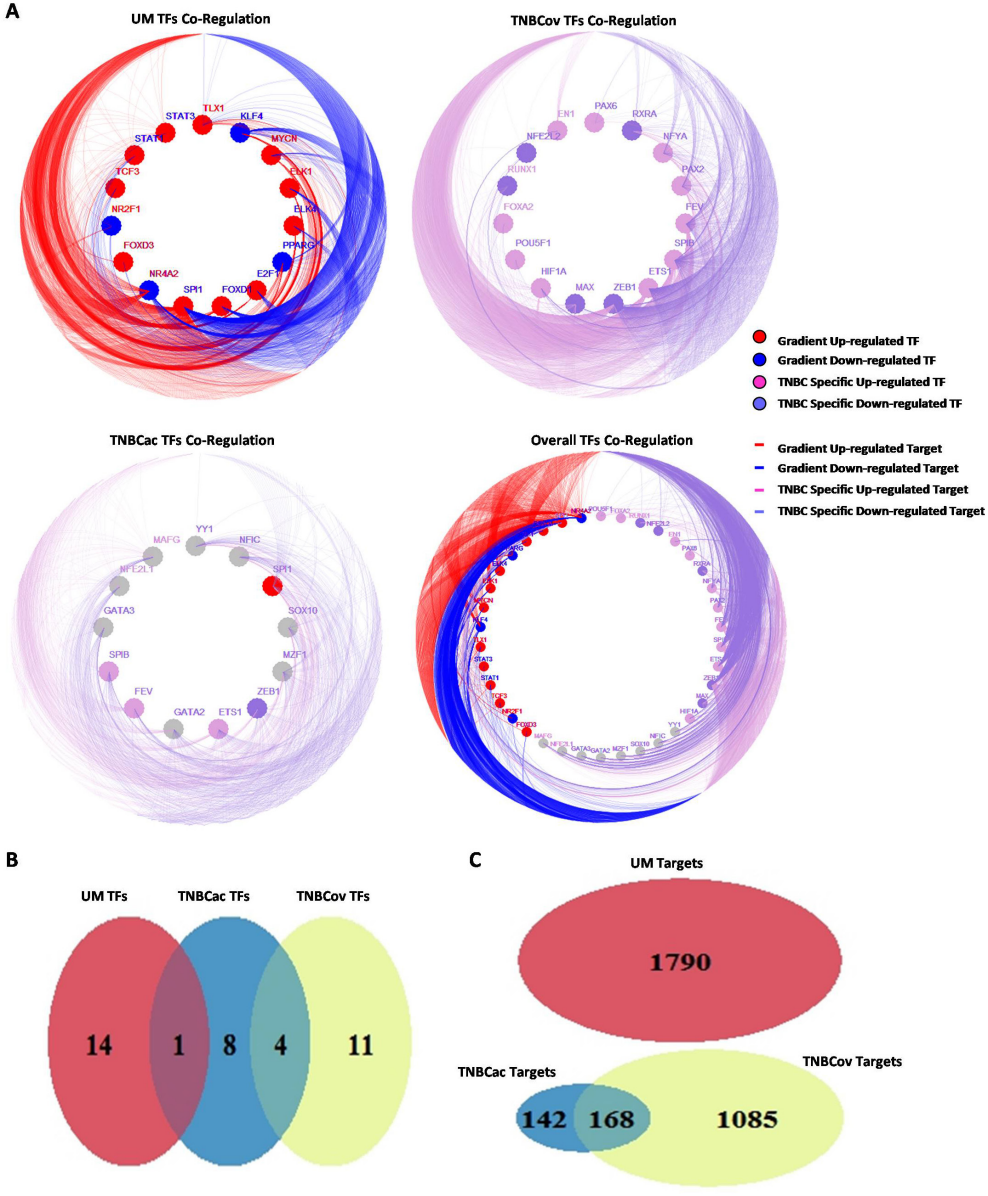
Pattern finding and sub-network construction **A.** TF co-regulation network in different regulation pattern (Solid circles distributed along inner ring stand for TFs, edges link the circles and outer ring stand for target genes of TFs, different color of circles and edges stand for different expression pattern of TFs and their targets); **B.** The overlap of TFs in different regulation pattern; **C.** The overlap of target genes in different regulation pattern

Co-regulation of all three patterns was shown in a CIRCOS-like plot (Figure 3A). Venn diagrams show overlaps of TFs and target genes in these three patterns (Figure 3B and 3C). Neither the TFs nor the target genes in TNBCov pattern had any overlap with the UM pattern, which is in accordance with their definitions. TFs in all three patterns were mostly unique, indicating that the patterns were tissue specific.

**Figure 4:**
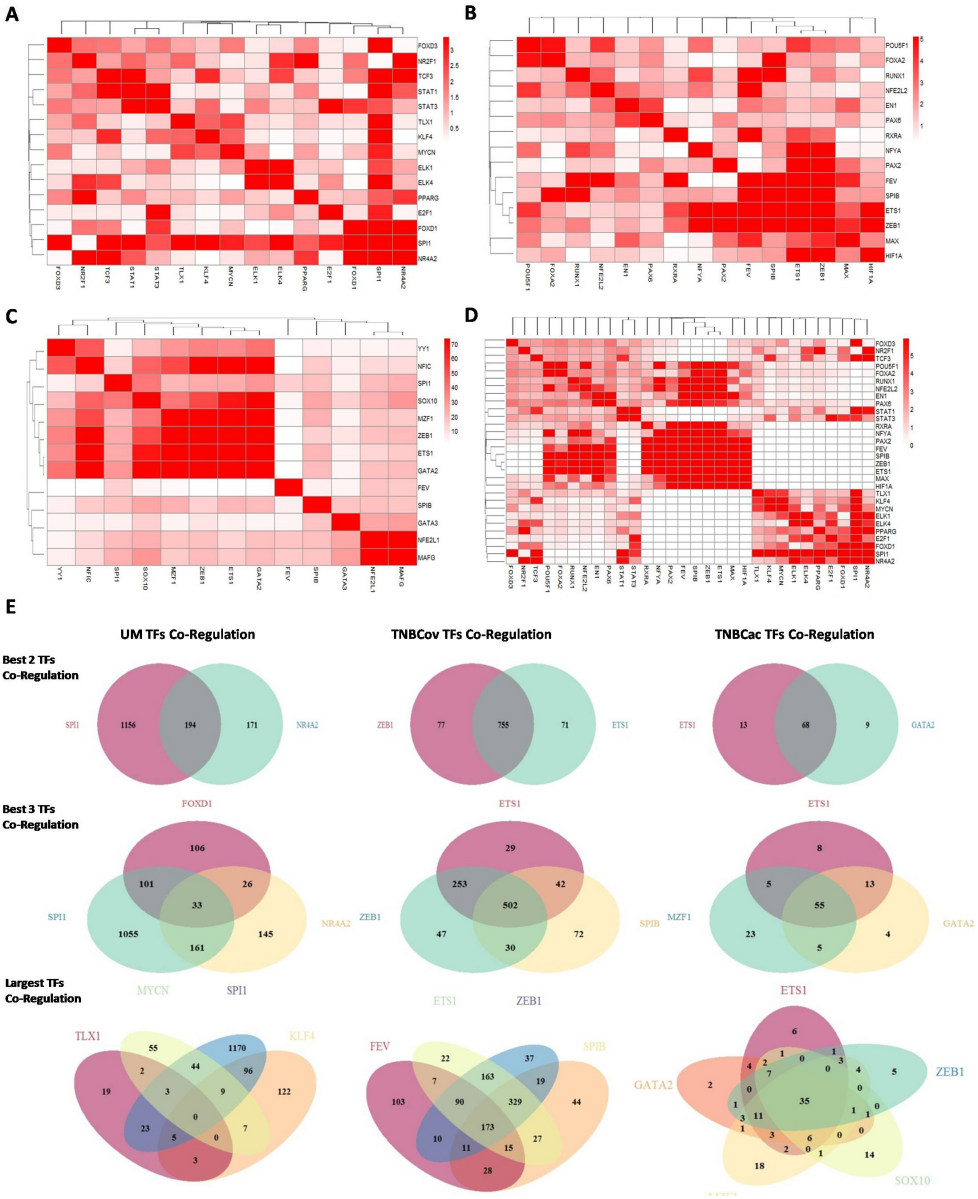
TF target profile similarity analysis and module finding **A.B.C.D.** Target profile similarity between the TFs in (UM pattern, TNBCov pattern, TNBCac pattern, All the above); **E.** Co-regulation modules found in UM pattern, TNBCov pattern, TNBCac pattern

TF target profile similarity analysis was performed to identify TFs co-regulation modules. Target profile similarity between TFs in the UM, TNBCov, and TNBCac pattern and all the three together was shown by consistency heatmap (Figure 4A–4D). TF co-regulation modules in different patterns were identified and summarized in Table 1. Representative two-TF co-regulation, three-TF co-regulation, and largest TFs co-regulation in different patterns were shown by a Venn diagram (Figure 4E).

**Table 1:** Co-Regulation TF modules in all three patterns

Co-Regulation in Different Pattern	Co-Regulation TF Groups
UM TFs Co-Regulation	1. FOXD1, SPI1, NR4A2 2. ELK1, ELK4, SPI1 3. TCF3, STAT1, STAT3, SPI1 4. TLX1, KLF4, MYCN, SPI1
TNBCov TFs Co-Regulation	1. FEV, SPIB, ETS1, ZEB1 2. ETS1, ZEB1, (RXRA, NFYA, PAX2, POU5F1) 3. POU5F1, FOXA2 4. RUNX1, NFE2L2, (NFYA, FEV) 5. EN1, PAXB, MAX
TNBCac TFs Co-Regulation	1. SOX10, M2F1, ZEB1, ETS1, GATA2 2. NFIC, SOX10, M2F1, ZEB1, ETS1, GATA2, (YY1, SPI1) 3. NFE2L1, MAFG, (GATA3, SPIB, FEV)

Of note, three patterns identified from our network analysis had very different topological differences. For the UM pattern, a gene was typically regulated by few TFs, but many TFs tend to share a common set of target genes for the TNBCac pattern.

### Functional analysis of TNBCac core genes and target genes in all three patterns

In the co-regulation analysis, we noticed that five TFs (SOX10, M2F1, ZEB1, ETS1, GATA2) shared most of their target genes together (35 target genes were identified to be regulated by all these five TFs in this pattern, Figure 4E, right down panel). Since the shared 35 genes (details listed in Supplementary Table 1) were insufficient to perform GO enrichment analysis, we explored the TF-target regulation network in TNBC, including genes that were not directly targeted but only a few steps away (described in Methods section). Finally 1,590 genes (including the initial 35 genes) were recruited for GO analysis. GO terms in three categories (response to stimulus, immune response and signal transduction) were found most significantly enriched in these 1,590 genes (Table 2). Stem cell related GO terms and epithelial-mesenchymal transition (EMT) related GO terms were also found significant (*p*<0.05) in our analysis, validating the previous findings that TNBC was associated with cancer stem cell (CSC) and EMT process [34, 35] (Supplementary Figure 2).

**Table 2:** GO enrichment analysis of the sub-network extended by 35 core genes (1590 genes included)

GOBPID	P-value	Count	Term
GO:0002376	7.34E-35	364	immune system process
GO:0001775	4.90E-31	184	cell activation
GO:0048518	5.98E-31	562	positive regulation of biological process
GO:0048584	1.62E-26	254	positive regulation of response to stimulus
GO:0048583	1.82E-26	429	regulation of response to stimulus
GO:0006955	4.21E-26	236	immune response
GO:0050896	4.29E-25	860	response to stimulus
GO:0045321	5.43E-25	140	leukocyte activation
GO:0002682	1.38E-24	206	regulation of immune system process
GO:0048522	2.95E-23	489	positive regulation of cellular process
GO:0007165	7.83E-22	621	signal transduction
GO:0023052	9.51E-22	669	signaling
GO:0044700	9.51E-22	669	single organism signaling
GO:0007154	3.19E-21	674	cell communication
GO:0046649	3.88E-21	119	lymphocyte activation
GO:0051716	4.09E-21	714	cellular response to stimulus
GO:0051239	4.66E-21	309	regulation of multicellular organismal process
GO:0006950	5.36E-21	437	response to stress
GO:0042110	1.12E-20	96	T cell activation

### TNBCac cores TFs are functionally essential in cancer cells

To test whether the predicted core genes were essential, we further conducted an integrated analysis combining CCLE expression data and Achilles shRNA screening data. Among the 5 core TFs identified in the largest TNBCac co-regulation module, ETS1 and GATA2 seemed to be not generally crucial in survival and growth of cancer cells (Supplementary Figure 3A), which may be due to nonlinear dose-dependence or insufficient shRNA interference efficiency. All MZF1 shRNAs, 4 out of 5 SOX10 shRNAs, and 2 of 3 ZEB1 shRNAs exhibited a strong effect on nearly all 212 cell lines (Supplementary Figure 3B), suggesting that these 3 TFs could be functionally essential in cancer cells.

**Figure 5:**
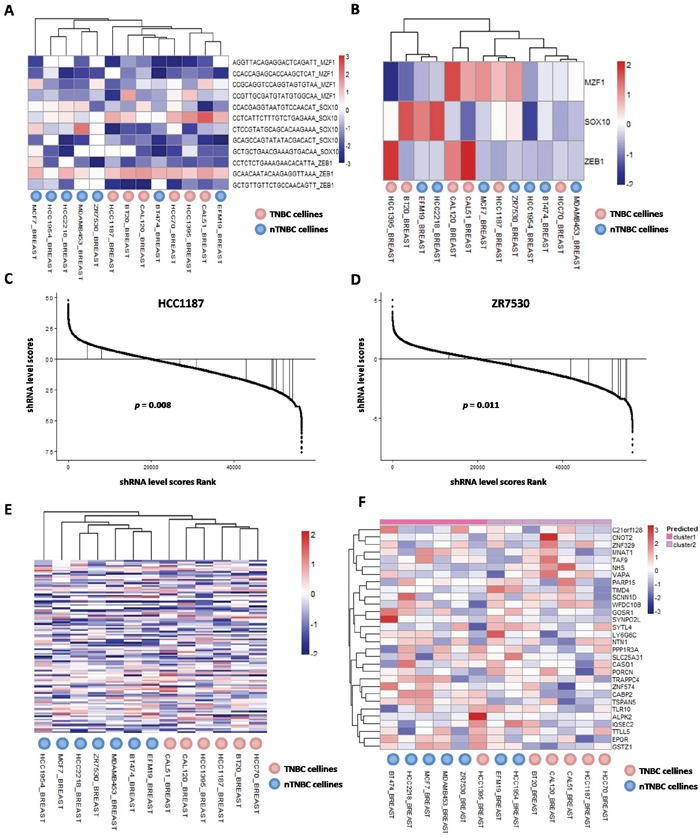
Essentialness evaluation of core TFs and their co-targeted genes in TNBCac pattern in breast cancer **A.** Heatmap and hierarchical clustering result of 13 Achilles breast cancer cell lines by siRNA scores of 3 Core TFs; **B.** Heatmap and hierarchical clustering result of 13 Achilles breast cancer cell lines by mRNA expression level of 3 Core TFs; **C.** Rank and siRNA scores of 3 Core TFs in HCC1187 cell line; **D.** Rank siRNA scores of 3 Core TFs in ZR7530 cell line; **E.** Heatmap and hierarchical clustering result of 13 Achilles breast cancer cell lines by siRNA scores of 35 Core co-targeted genes; **F.** Heatmap and hierarchical clustering result of 13 Achilles breast cancer cell lines by mRNA expression level of 35 Core co-targeted genes

Furthermore, clustering 13 breast cancer cell lines with shRNA scores of MZF1, SOX10 and ZEB1, could roughly distinguish TNBC cell lines from nTNBC cell lines (Figure 5A). Of note, only two nTNBC cell lines BT474 and EFM19 were clustered together with TNBC cell lines, whereas all TNBC cell lines were clustered in the same group. In contrast, analysis of the expression data of these TFs only was unable to reproduce the clusters (Figure 5B), indicating that our network analysis provides significant new biological insights of these TFs. Representative shRNA score distributions of MZF1, SOX10 and ZEB1 were displayed in HCC1187 (Figure 5C) and ZR7530 (Figure 5D).

The 35 core target genes were also investigated. Generally, these genes are functionally essential in cancer cells (Supplementary Figure 3C), and their shRNA scores could precisely distinguish TNBC cell lines from nTNBC cell lines without any mismatch (Figure 5E). The expression data of these genes had a moderate accuracy in discriminating TNBC from nTNBC cells (Figure 5F), suggesting that the difference of these target genes in TNBC and nTNBC was mainly at expression level.

### *In vitro* validation of the core TFs' essentialness and regulatory role in TNBC

**Figure 6:**
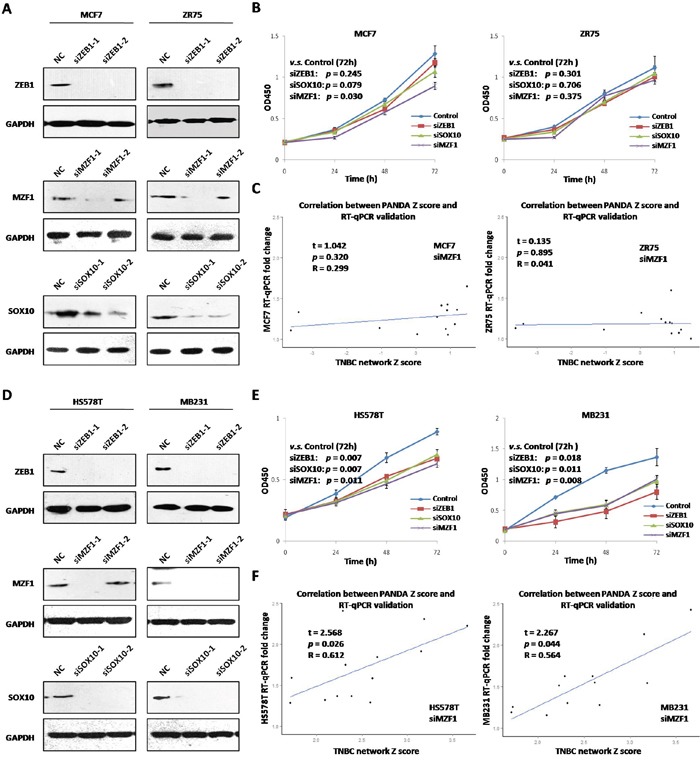
Essentialness validation of core TFs in breast cancer cell lines **A.** Silencing of ZEB1, MZF1, SOX10 by two siRNAs in nTNBC cells (MCF-7 and ZR75); **B.** Cell proliferation cure after silencing of ZEB1, MZF1, SOX10 in nTNBC cells; **C.** Correlation between predicted TF-target Z-score and target gene expression fold change after silencing of MZF1 in nTNBC cells; **D.** Silencing of ZEB1, MZF1, SOX10 by two siRNAs in TNBC cells (HS578T and MB231); **E.** Cell proliferation cure after silencing of ZEB1, MZF1, SOX10 in TNBC cells; **F.** Correlation between predicted TF-target Z-score and target gene expression fold change after silencing ofMZF1 in TNBC cells

To validate the essentialness of the core TFs (MZF1, SOX10 and ZEB1) in different breast cancer cell lines, four TNBC and four nTNBC cell lines were used for CCK8 cell proliferation analysis. Two different siRNAs of each core TFs were transfected in all eight cell lines (Figure 6A&6D, Supplementary Figure 4A), and the ones with better interfering efficiency were used for subsequent CCK8 and RT-qPCR analysis. After silencing of each core TFs, TNBC but not nTNBC cell proliferation rate changed significantly (Figure 6B and 6C, Supplementary Figure 4B, the only exception was siMZF1 in MCF7 cells). Thus our results, both *in silico* and *in vitro*, indicated that these 3 TFs were functionally essential for TNBC but not for nTNBC cell proliferation.

To validate the TF-target correlation of core TFs in breast cancer cell lines, 13 of the 35 core target genes were assessed by RT-qPCR after silencing of each core TFs in two nTNBC cells (MCF-7/ZR75) and TNBC cells (HS578T/MB231). The expression fold change of the target genes after MZF1 silencing in nTNBC cells was not significantly correlated with predicted nTNBC MZF1-target edge Z-scores (MCF-7, R=0.299, *p*=0.320; ZR75, R=0.041, *p*=0.895, Figure 6E and 6F). However, fold change in TNBC cells was significantly correlated with predicted TNBC MZF1-target edge Z-scores (HS578T, R=0.612, *p=*0.026; MB231, R=0.564, *p*=0.044). Silencing of SOX10 and ZEB1 also achieved similar results (Supplementary Figure 5), suggesting that regulatory relationships between these 3 TFs and the core target genes were TNBC specific as predicted.

### TNBCac pattern recapitulates TNBC status and is associated with survival

The 35 core genes and their co-regulators (not only TFs in TNBCac patterns) were collected as a novel gene signature, and clinical application of this gene signature was explored in several datasets.

**Figure 7:**
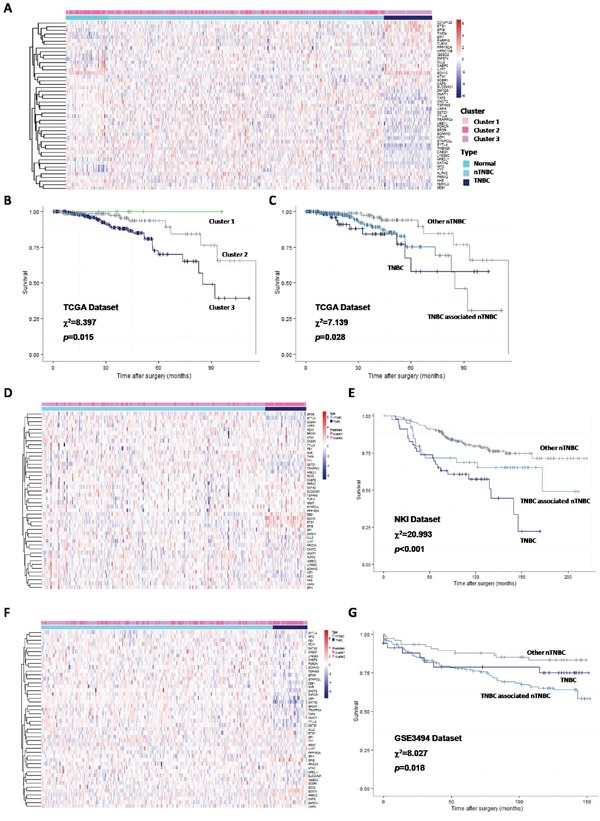
Clustering breast cancer patients by 35 core genes and their regulators, and survival analysis **A.** Heatmap and hierarchical clustering result of TCGA breast cancer patients by 35 core genes and their regulators, 3 subgroups were isolated according to the hierarchical tree; **B.** Kaplan-Meier curve of DMFS in TCGA breast cancer patients, grouped by clustering result; **C.** Kaplan-Meier curve of DMFS in TCGA breast cancer patients, all patients were grouped to TNBC, nTNBC with same core expression profile with TNBC, and other nTNBC; **D.** Heatmap and k-means clustering result of validating DATASET1 by 35 core genes and their regulators; **E.** Kaplan-Meier curve of DMFS in validating DATASET1, all patients were grouped to TNBC, nTNBC with same core expression profile with TNBC, and other nTNBC; **F.** Heatmap and k-means clustering result of validating DATASET2 by 35 core genes and their regulators; **G.** Kaplan-Meier curve of DMFS in validating DATASET2, all patients were grouped to TNBC, nTNBC with same core expression profile with TNBC, and other nTNBC

Clustering result of TCGA breast cancer patients by these genes had high accordance with the NORM-nTNBC-TNBC classification (Figure 7A). Nearly all TNBC were classified into the same subgroup (Cluster 3) which has the worst prognosis, and the only two TNBC patients classified to the other subgroup (Cluster 1) were still alive till last follow-up (Figure 7B), suggesting the tumor in these patients was less aggressive.

We further stratified nTNBC patients into two subgroups according to similarity with the TNBCac pattern. Strikingly, the subgroup that more resembles TNBC turned out to have a worse prognosis than the other subgroup (Figure 7C), suggesting that the TNBCac signature can also be used as a guide to identify more aggressive nTNBC tumors. To test if this prediction is robust, we applied the same analysis to two independent breast cancer datasets (NKI and GSE3494), and achieved similar results (Figure 7D–7G).

## DISCUSSION

Although the molecular traits of breast cancer have been discussed in previous reports, studies addressing the regulatory spectrums of breast cancer subtypes were rare [10–12]. Using network topologies and gene expression differences among NORM, nTNBC and TNBC tissues, we distinguished three different TF-gene regulatory patterns, which reflected three different biological regulatory modes. The TNBCac pattern exhibited a highly significant TF-TF co-regulatory mode. On the contrary, the TFs involved in UM pattern showed a very weak relationship with each other. Thus TNBC may directly originate from NORM instead of nTNBC. This hypothesis is consistent with the fact that transition from nTNBC to TNBC was barely observed in clinical patients [3]. Considering that TF-TF co-regulation was much more significant in TNBCac than in TNBCov, the process of initiating TNBC would more possibly be TF activation driven than TF overexpression driven.

A core co-regulatory module with 5 TFs and 35 co-targeted genes was identified in TNBCac pattern, and these genes were positioned in the network which is highly associated with response to stimulus, immune response and signal transduction. For response to stimulus, seven related GOs were found in the top20 significant GOs. Previous studies also indicated that stimulus response was highly associated with EMT process, and environmental stress strongly affected the metabolic activity in breast cancer cells [34, 35]. For immune response, six related GOs were found in the top20 significant GOs. Immune response is complicated and could affect carcinogenesis by inflammation [36, 37], autoimmune [38] and immune escape [39] in TNBC. Our findings further indicated that this field was remarkable. For signal transduction, four related GOs were found in the top20 significant GOs. Many signaling pathways such as MAPK, Wnt, and Erk, were found crucial in TNBC [40–43], which could validate our findings.

Furthermore, the essentialness of these genes in cancer cell survival was investigated, especially the core 5 TFs in TNBCac pattern. MZF1, SOX10 and ZEB1 shRNAs displayed strong effect on survival of cancer cells. However, ETS1 and GATA2 seemed to be less crucial in the same system, which might be due to nonlinear dose-dependence or insufficient shRNA interference efficiency. When ruling out the two puzzling TFs, 3 core TFs in the module could still clearly distinguish TNBC cells from nTNBC cells by their essentialness scores, The expression of the 3 core TFs could not distinguish TNBC from nTNBC like their essentialness scores, suggesting that the importance of these 3 TFs in TNBC would mainly due to possible activation process (e.g. post-translational modification) but not the change at expression level.

MZF1 was found crucial in osteopontin-driven MSC-to-CAF transformation, which promoted tumor growth in a microenvironment dependent manner [44]. MZF1 is also a regulator of ERCC1 and affects DNA damage/repair pathway, which is essential in chemo-resistance [45]. SOX10 was reported to be preferentially overexpressed in TNBC [46] and appeared to be a part of a highly coordinated transcriptional program characteristic for basal-like features [47]. As a well-studied TF, ZEB1 was highly involved in EMT process and also reported promoting migration in TNBC cells by regulating androgen receptor (AR) [44]. Additionally, it could also enhance tumorigenicity and breast cancer cell plasticity [48]. The 3 core TFs were all found to influence TNBC crucially, but their co-activation was not reported. Our results suggested exploring them as a whole module propounds a further investigation of their co-regulation and co-targeting profile.

Additionally, the core targets genes showed a distinct discrimination between TNBC and nTNBC, not only at essentialness score level but also at expression level, which confirmed our hypothesis that the core 3 TFs promoted TNBC related biological process by regulation of the expression of the core target 35 genes.

Classifying breast cancer by only three markers (ER, PgR, HER2) is rough, and the definition of TNBC did not seem to be rigorous [1, 8, 13]. Recently, development of new technology and algorithm makes it possible to divide breast cancer patients to subgroups more scientifically [1, 13]. Focusing on the heterogeneity of TNBC, many sub-classification systems were developed. However, the heterogeneity of nTNBC was not so appealing even though the prognosis of which varies much more [1]. By clustering patients with our own signature based on the core module found in TNBC, nearly all TNBC patients were clustered into the same subgroup while some nTNBC patients were also clustered with TNBC. In other words, we identified a TNBC-like nTNBC subgroup, which also showed a similar prognosis as TNBC. Furthermore, this classification system was applied in three different cohorts with more than 1000 patients, which conferred this signature close to clinical translation. Compared with the most widely used breast cancer molecular classification system PAM50, which included genes with certain functions in breast cancer [49, 50], our signature focused mainly on translational regulatory features in TNBC and included a whole co-regulatory module. There is little overlap in candidate genes between PAM50 and our signature, so that our signature would be a very important complement to PAM50.

In summary, we established TF-gene regulatory networks in TNBC, found three different patterns, and identified a core TF co-regulatory module comprised of 5 TFs and 35 target genes. These core genes exhibited strong effect on cancer cell survival and growth. Furthermore, the 3 core TFs could distinguish nTNBC cell lines from TNBC cell lines by their “essentialness profile”. The 35 core target genes could distinguish nTNBC cell lines from TNBC cell lines by both expression profile and “essentialness profile”. The overall expression profile of the core targets and their regulators identified a TNBC-like subgroup of nTNBC, whose prognosis was more analogous to TNBC than to other nTNBC, suggesting a promising clinical application perspective. Generally, our results demonstrated a novel and biologically reasonable view to TNBC and enabling nTNBC subtype re-classification based on a TNBC-associated manner. In addition, the methods we described here are not only limited to the analysis of TNBC but also are generalizable to other complicated diseases that demonstrate subtype-specific characteristics, especially those without well-defined molecular targets.

## MATERIALS AND METHODS

### Data acquisition and preparation

Microarray gene expression data from 63 normal breast (NORM) tissue samples, 445 non-triple-negative breast cancer (nTNBC) tissue samples and 89 triple-negative breast cancer (TNBC) tissue samples were downloaded from TCGA (http://cancergenome.nih.gov/) for primary analysis and TF-targets network construction [25, 26]. Datasets NKI (http://ccb.nki.nl/data/) and GSE3494 (http://www.ncbi.nlm.nih.gov/geo/) were used for validation [31, 32]. Robust Multichip Average (RMA) [51] method was used for normalization.

Position weight matrix (PWM) data of 130 core TF binding sequence motifs in vertebrates were downloaded from JASPAR database [52]. Each motif matrix is used to scan the entire human genome and a threshold value of *p<*10^−5^was used to determine motif sites. For each motif, we determined its target genes as those whose promoter regions, defined as [−750, 250] base-pairs flanking their transcriptional start sites (TSS), contain at least one motif site. For protein-protein interactions (PPI), we used a publicly available dataset as an estimate [53].

The Cancer Cell Line Encyclopedia (CCLE) (http://www.broadinstitute.org/ccle) database and Achilles database (http://www.broadinstitute.org/achilles) [27–30] were downloaded. 212 cell lines (13 breast cancer cell lines included) with both mRNA expression data and shRNA level scores data were integrated for subsequent analyses.

### Network construction and comparison

The PANDA software (http://sourceforge.net/projects/panda-net/) was used for network construction [19, 23, 24]. Networks of NORM, nTNBC and TNBC were constructed by integrating the corresponding TCGA expression, TF motif and PPI data (update parameter α=0.25). A cutoff of FDR adjusted *p<*0.05 was used to determine significant edges.

### TFs co-regulation analysis and target profile merging

The hypergeometric distribution model was applied to evaluate the overlap between target genes shared by different TFs. All significant 2-TFs co-regulation genesets were mutually merged for intersections. Genes intersected from four or three 2-TFs co-regulation genesets were defined as 4-TFs or 3-TFs co-regulation genesets, respectively, and were then evaluated with the same hypergeometric distribution model. Larger (5-8-TFs) gene sets were gained by a next merging step with all significant 4-TFs co-regulation genesets.

### Core network extension and GO enrichment analysis

The core 35 target genes were reset to TNBC network and their neighbors in this network were looked up by a “network walking” method as described in the following. All TFs regulated more than 10 of these 35 genes were selected as intermediators, while all genes co-regulated by more than 20 intermediators were chosen as neighbors of these 35 genes and used for gene ontology (GO) enrichment analysis (biological process [BP] category, performed by R packages). The hypergeometric distribution model along with a false discovery rate (FDR) adjustment was used for significance evaluation.

### Cell culture, small interfering RNAs transfection and CCK8 analysis

Breast cancer cell lines MCF7, ZR75, MDA435, MDA453, MB231, BT20, HS578T, and HCC1937 were purchased from American Type Culture Collection (ATCC) and maintained in standard conditions. Transfection was performed with Lipofectamine 2000 (Invitrogen, Carlsbad, CA) according to the manufacturer’s protocol. Targeted sequences for small interfering RNA (siRNA)-induced silencing were all listed in Supplementary Table 2.

Cell suspension (100 μL/well) was inoculated in a 96-wellplate, pre-incubated in a 37°C humidified incubator (5% CO2). After each of the 0, 24, 48, and 72 h time points, 10 μL of the CCK8 reagent from Sigma (St.Louis, MO) was added to each well of the corresponding plate. The plate was incubated for two additional hours and the 450nm absorbance was measured.

### Western blot and real-time RT–qPCR

Cell total RNA was extracted with Trizol reagent (Invitrogen) and cDNA was synthesized from at least 3μg of total RNA using oligo (dT) and random hexamer primers. All primers (synthesized by GenePharma) used for RT–qPCR were listed in the Supplementary Table 3, and qPCR settings were 94°C for 2 min followed by 35 cycles of 94°C 15 s, 56°C 20 s and 72°C 30 s and then followed by 72°C for 2 min.

Cell total proteins were obtained by homogenization in 2× loading buffer, resolved by sodium dodecyl sulfate–polyacrylamide gel electrophoresis and subjected to western blot with corresponding antibodies. Anti-MZF1 and anti-SOX10 antibodies were purchased from Cell Signaling Technology (Beverly, MA). Anti-ZEB1 antibody was purchased from Abcam (Cambridge, MA).

### Patients clustering and survival analysis

All patients were clustered by a k-means method, where k was set to 3 (NORM, nTNBC and TNBC) or 2 (when only nTNBC and TNBC were considered). Genes were clustered by hierarchical clustering. Expression levels of all genes were normalized by row before heatmap visualization. Kaplan-Meier analysis and Log-rank test were used to evaluate survival rates.

### Statistical analysis

All statistical tests were 2-sided and performed using R 3.1.2 software (www.r-project.org). *p<*0.05 was considered statistically significant unless otherwise mentioned. A cutoff value of FDR<0.1 was used for multiple comparisons. R packages ggplot2, VennDiagram, and pheatmap were used for data visualization; Mygene, GEOquery and GOstats were used for gene symbol mapping and GO enrichment. R packages survival and MASS were used for survival analysis.

## SUPPLEMENTARY MATERIALS FIGURES AND TABLES


