# Supplementary material for: Gene regulatory pattern analysis reveals essential role of core transcriptional factors’ activation in triple-negative breast cancer
Source: Oncotarget. 2017 Feb 27;8(13):21938–53. doi: 10.18632/oncotarget.15749 (PMC5400636; doi:10.18632/oncotarget.15749)
Supplement: Supplementary file 1 [file oncotarget-08-21938-s001.pdf]

# Gene regulatory pattern analysis reveals essential role of core transcriptional factors' activation in triple-negative breast cancer

## SUPPLEMENTARY MATERIALS

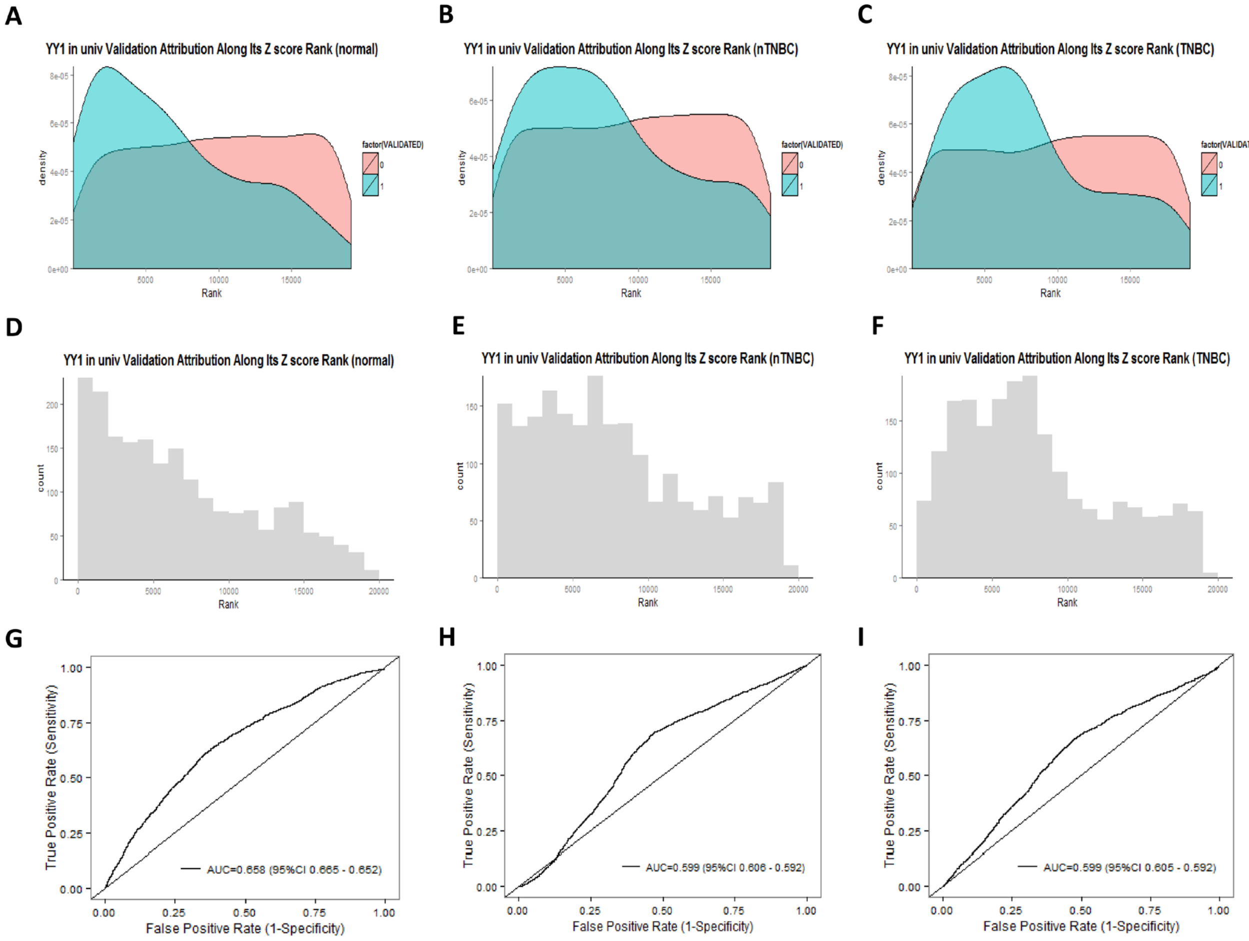


**Supplementary Figure 1: Gene regulatory network construction and validation (YY1). A.B.C.** Density distribution of edges aligning by Z score rank, grouped by ENCODE ChIP-seq data (normal, nTNBC, TNBC); **D.E.F.** Histogram of ENCODE edges aligning by Z score rank of PANDA network (normal, nTNBC, TNBC); **G.H.I.** ROC curve of ENCODE edges aligning by Z score rank of PANDA network (normal, nTNBC, TNBC).

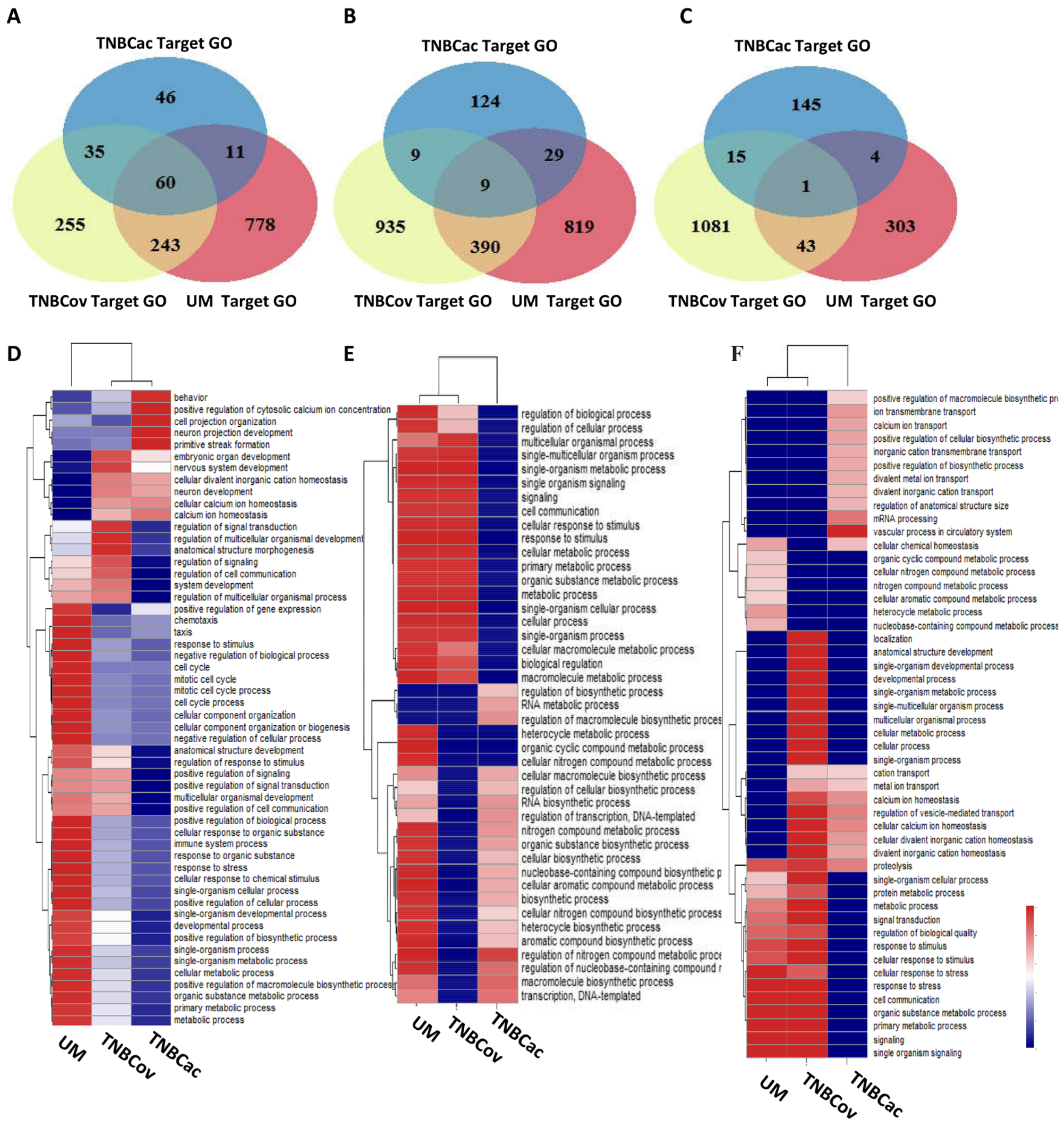


**Supplementary Figure 2: GO analysis of target genes in pre-defined patterns.** **A.B.C.** The overlap of GO enrichment in different regulation pattern (All targets, Top 5 2TF module targets, Top 5 3TF module targets); **D.E.F.** Clustering GO enrichment of target genes in different regulation pattern (All targets, Top 5 2TF module targets, Top 5 3TF module targets).

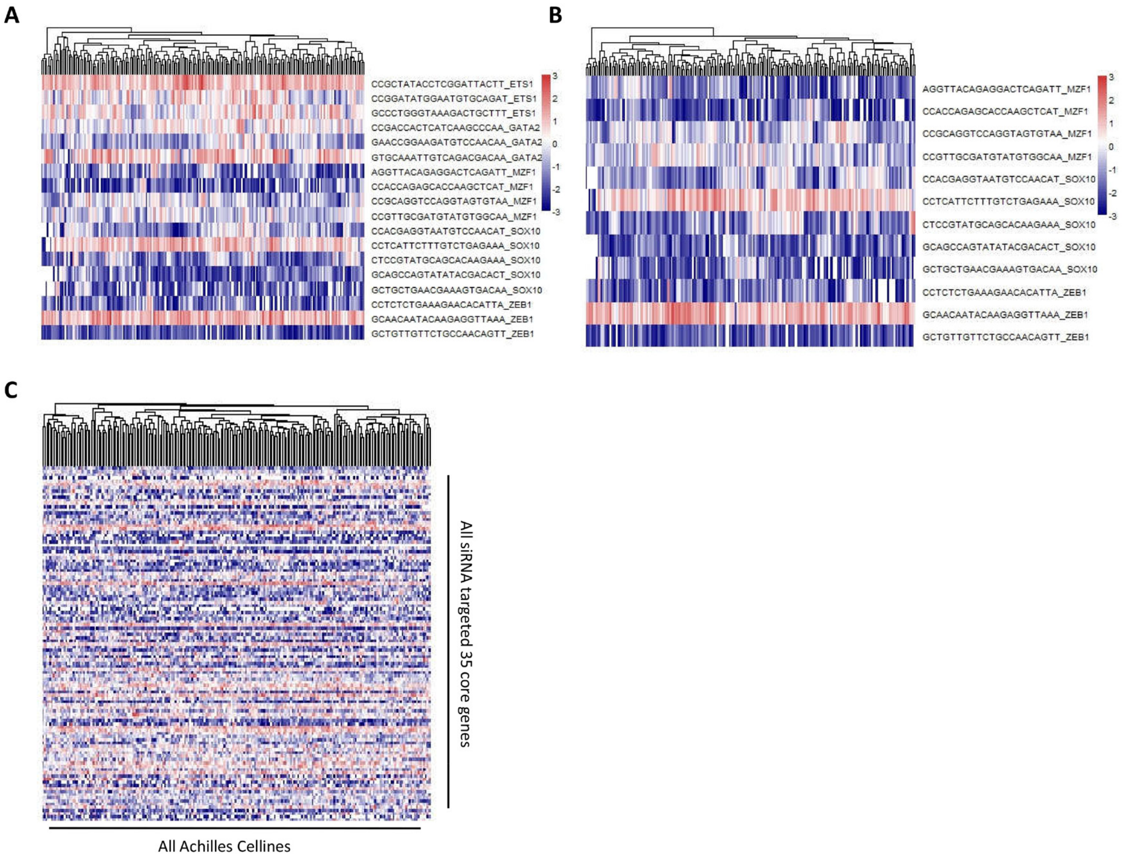


**Supplementary Figure 3: Essentialness evaluation of Core TFs and their co-targeted genes in TNBCac pattern. A.** Heatmap and hierarchical clustering result of 212 Achilles cancer cellines by siRNA scores of 5 Core TFs; **B.** Heatmap and hierarchical clustering result of 212 Achilles cancer cellines by siRNA scores of 3 Core TFs; **C.** Heatmap and hierarchical clustering result of 212 Achilles cancer cellines by siRNA scores of 35 Core co-targeted genes.

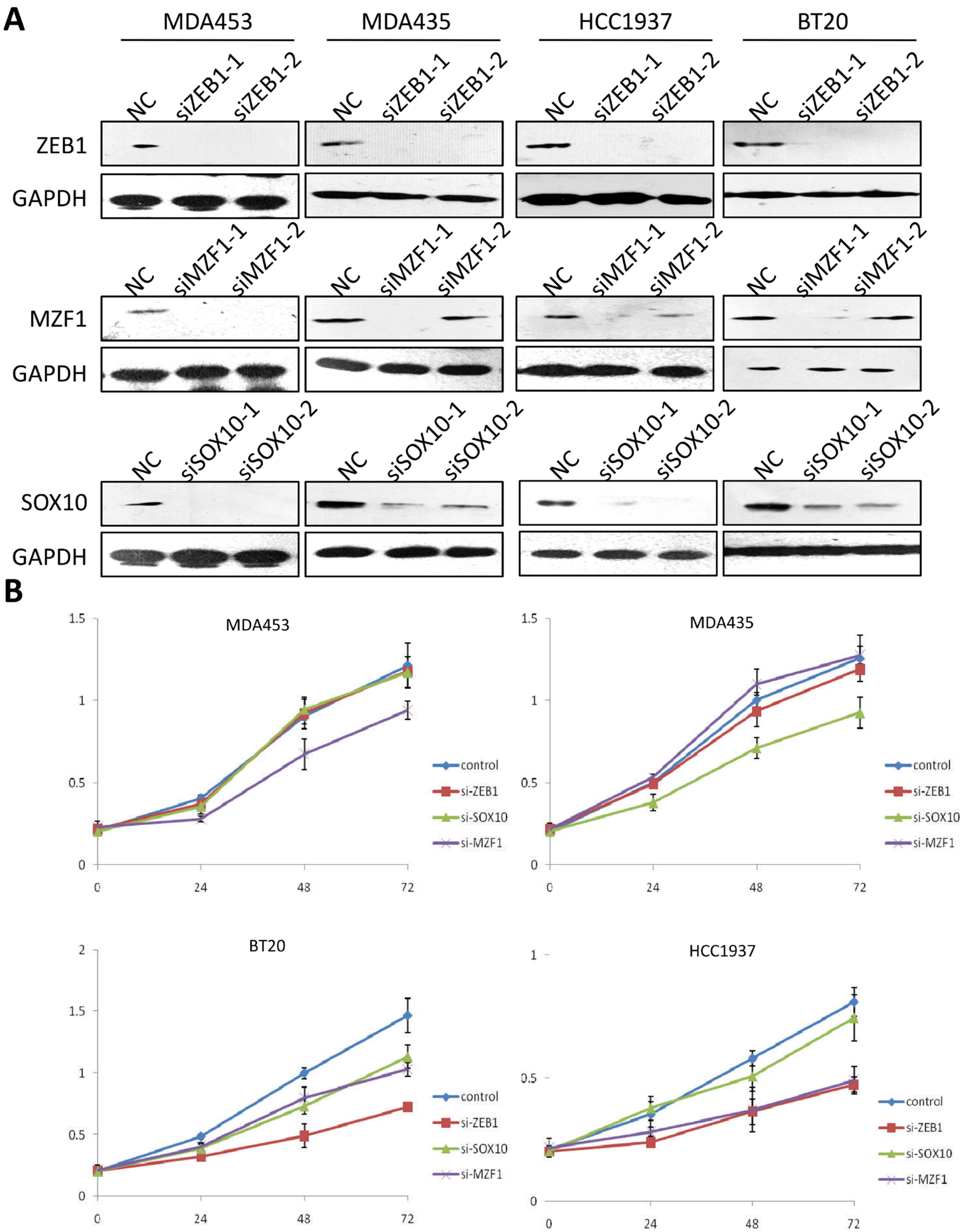


**Supplementary Figure 4: Essentialness validation of core TFs in breast cancer cell-lines. A.** Knockdown of ZEB1, MZF1, SOX10 by two siRNAs in nTNBC cells (MDA453 and MDA435) and TNBC cells (BT20 and HCC1937); **B.** Cell proliferation cure after silencing of ZEB1, MZF1, SOX10 in nTNBC cells (MDA453 and MDA435) and TNBC cells (BT20 and HCC1937).

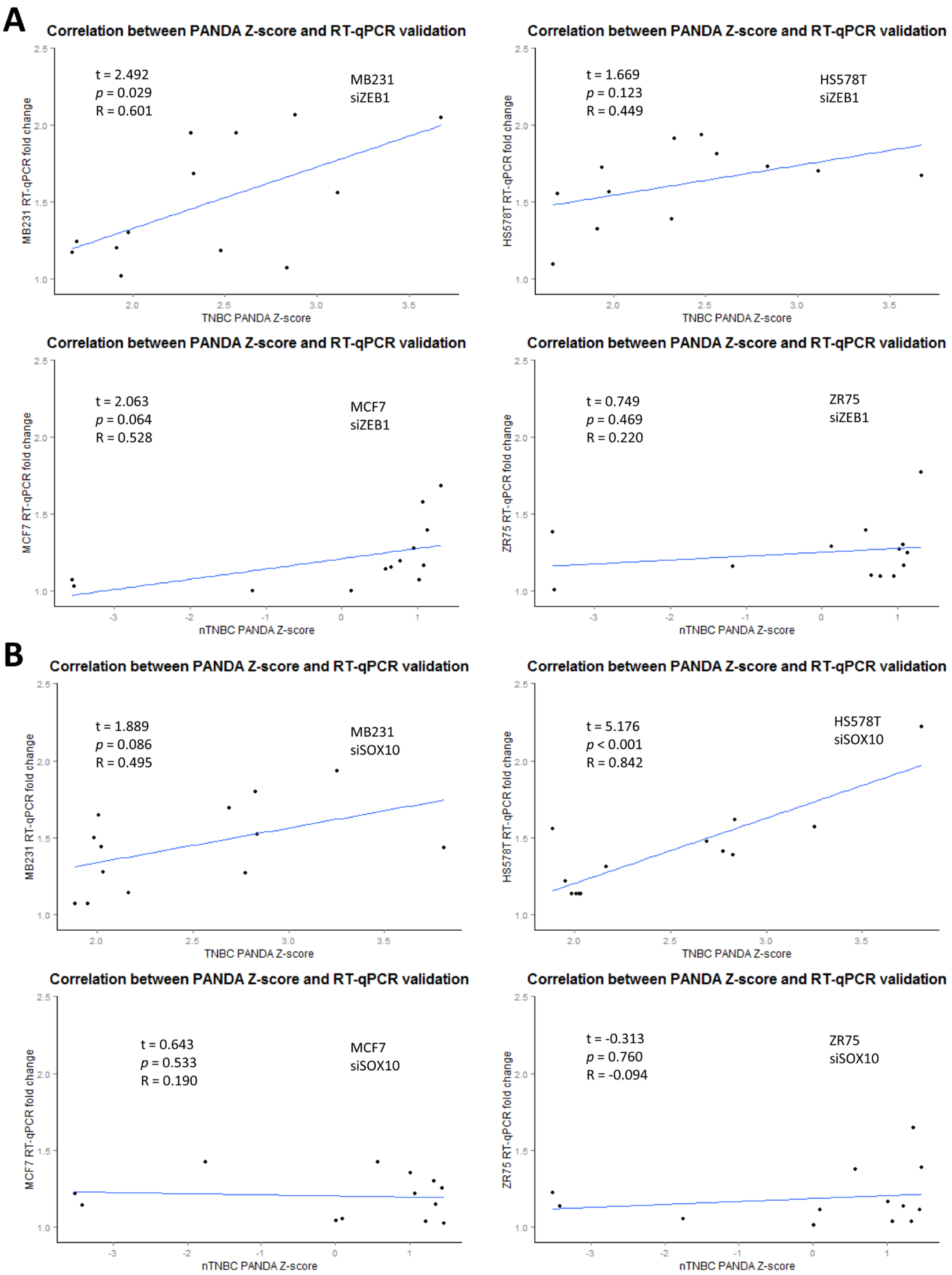


**Supplementary Figure 5: TF-target correlation validation of core TFs in breast cancer cell-lines. A.** Correlation between predicted TF-target Z-score and target gene expression fold change after MZF1 in nTNBC cells (MCF-7 and ZR75) and TNBC cells (HS578T and MB231); **B.** Correlation between predicted TF-target Z-score and target gene expression fold change after MZF1 in nTNBC cells (MCF-7 and ZR75) and TNBC cells (HS578T and MB231).

**Supplementary Table 1: Details of the core 35 target genes**

| query | name | go.BP.id | go.CC.id | go.MF.id |
|---|---|---|---|---|
| ALPK2 | alpha kinase 2 | GO:0006468 | GO:0005654 | NA |
| C21orf128 | UMODL1 Antisense RNA 1 | NA | NA | NA |
| CABP2 | calcium binding protein 2 | GO:0007165 | NA | GO:0005509 |
| CASQ1 | calsequestrin 1 | NA | NA | GO:0005509 |
| CNOT2 | CCR4-NOT transcription complex subunit 2 | NA | NA | NA |
| EPOR | erythropoietin receptor | NA | NA | NA |
| FER1L3 | Myoferlin | NA | NA | NA |
| GOSR1 | golgi SNAP receptor complex member 1 | NA | NA | NA |
| GSTZ1 | glutathione S-transferase zeta 1 | NA | NA | NA |
| IL1F7 | Interleukin 37 | NA | NA | NA |
| IQSEC2 | IQ motif and Sec7 domain 2 | NA | GO:0005737 | GO:0005086 |
| LY6G6C | lymphocyte antigen 6 complex, locus G6C | NA | NA | NA |
| MLL2 | Lysine (K)-Specific Methyltransferase 2D | NA | NA | NA |
| MNAT1 | MNAT CDK-activating kinase assembly factor 1 | NA | NA | NA |
| NHS | NHS actin remodeling regulator | NA | NA | NA |
| NTN1 | netrin 1 | NA | NA | GO:0005515 |
| PARP15 | poly(ADP-ribose) polymerase family member 15 | NA | GO:0005634 | GO:0003950 |
| PORCN | porcupine homolog (Drosophila) | NA | NA | NA |
| PPP1R3A | protein phosphatase 1 regulatory subunit 3A | GO:0005977 | GO:0016021 | NA |
| SCNN1D | sodium channel, non-voltage gated 1 delta subunit | NA | NA | |
| SLC25A31 | solute carrier family 25 (mitochondrial carrier; adenine nucleotide translocator), member 31 | NA | NA | NA |
| SYNPO2L | synaptopodin 2-like | NA | NA | GO:0003779 |
| SYTL4 | synaptotagmin like 4 | NA | NA | NA |
| TAF9 | TATA-box binding protein associated factor 9 | NA | NA | NA |
| TIMD4 | T-cell immunoglobulin and mucin domain containing 4 | NA | GO:0016021 | NA |
| TLR10 | toll-like receptor 10 | NA | NA | GO:0004888 |
| TMEM26 | transmembrane protein 26 | NA | GO:0016021 | NA |
| TRAPPC4 | trafficking protein particle complex 4 | NA | NA | NA |
| TSPAN5 | tetraspanin 5 | NA | NA | GO:0019899 |
| TTLL5 | tubulin tyrosine ligase like 5 | NA | NA | NA |
| UBE1L | Ubiquitin-Like Modifier Activating Enzyme 7 | NA | NA | NA |
| VAPA | VAMP associated protein A | NA | NA | NA |
| WFDC10B | WAP four-disulfide core domain 10B | GO:0010466 | GO:0005576 | GO:0030414 |
| ZNF329 | zinc finger protein 329 | NA | GO:0005634 | NA |
| ZNF574 | zinc finger protein 574 | NA | GO:0005634 | NA |

**Supplementary Table 2: Sequence information of siRNAs**

| Name | Sequence | |
|---|---|---|
| | sense (5'-3') | antisense (5'-3') |
| MZF1-homo-297 | GCCUGUCAUGGUGAAGCUATT | UAGCUUCACCAUGACAGGCTT |
| MZF1-homo-2400 | CCAGAGCACCAAGCUCAUUTT | AAUGAGCUUGGUGCUCUGGTT |
| SOX10-Homo-758 | CCGUAUGCAGCACAAGAAATT | UUUCUUGUGCUGCAUACGGTT |
| SOX10-Homo-1078 | GGAAGCCUCACAUCGACUUTT | AAGUCGAUGUGAGGCUUCCTT |
| ZEB1-Homo-1588 | GCUACUGGAGAUGGCAAUUTT | AAUUGCCAUCUCCAGUAGCTT |
| ZEB1-Homo-1936 | GGAUCAACCACCAAUGGUUTT | AACCAUUGGUGGUUGAUCCTT |

**Supplementary Table 3: Primer information for TF-target qPCR validation**

| Gene Symbol | PrimerName | PrimerSeq | PrimerSize | Tm °C | Position | Length Of Product |
|---|---|---|---|---|---|---|
| CABP2 | Forward Primer | GAGACGGCAGACATGATCGG | 20 | 62.4 | 439-458 | 165 |
| | Reverse Primer | CACGTCCTGGAGGATCTCG | 19 | 61.2 | 603-585 | |
| CASQ1 | Forward Primer | GGACACCCAAGTCAGGGGTA | 20 | 62.7 | 77-96 | 145 |
| | Reverse Primer | GGTTCATGGTAGAGGAGTGCC | 21 | 61.9 | 221-201 | |
| ALPK2 | Forward Primer | TGCTGTCTATCAAATCTCGGCT | 22 | 61.2 | 246-267 | 75 |
| | Reverse Primer | GAGCACTCAACCTCAACGGA | 20 | 61.8 | 320-301 | |
| TAF9 | Forward Primer | AGAGAAGAGCATAAAAGAGGCCA | 23 | 61.1 | 798-820 | 140 |
| | Reverse Primer | TCTGCCGCCGTTTCTTAAACA | 21 | 62.5 | 910-890 | |
| PARP15 | Forward Primer | AGCCGCCAGGAATTTTTCCA | 20 | 62.4 | 13-32 | 83 |
| | Reverse Primer | AGCACGAGGTAGCACAGGA | 19 | 62.6 | 257-239 | |
| PPP1R3A | Forward Primer | GTTTCTCCCCTCAACCAAGTAG | 22 | 60 | 107-128 | 157 |
| | Reverse Primer | GCACTCGGTAATTCCCAGCAA | 21 | 62.7 | 263-243 | |
| SCNN1D | Forward Primer | AGGAGGCTCACCTGGTCAT | 19 | 62 | 73-91 | 191 |
| | Reverse Primer | TGTATCGGGCCAGAGAGTAGG | 21 | 62.2 | 263-243 | |
| SYTL4 | Forward Primer | CAACACTACAGTGATCGGACC | 21 | 60.1 | 166-186 | 84 |
| | Reverse Primer | ACAACCCCGACAAGTATTGGT | 21 | 61.4 | 249-229 | |
| TLR10 | Forward Primer | AGGTTTGAGTGGGGCAAAAAT | 21 | 60.4 | 441-461 | 189 |
| | Reverse Primer | CCATCACGCAAAAGAACCCAG | 21 | 61.8 | 629-609 | |
| TMEM26 | Forward Primer | ATGGAGGGACTGGTCTTCCTT | 21 | 62 | 1-21 | 93 |
| | Reverse Primer | CTTCACCTCGGTCACTCGC | 19 | 62.4 | 93-75 | |
| TRAPPC4 | Forward Primer | AAGCTGGCGGCTTGATTTAC | 20 | 61 | 32-51 | 117 |
| | Reverse Primer | CAACCAACACACGCTCATCG | 20 | 61.9 | 148-129 | |
| TTLL5 | Forward Primer | TATTCCATGCCGACGCTATTC | 21 | 60 | 128-148 | 97 |
| | Reverse Primer | CGACTGTCCGTTCGTACAATCT | 22 | 62 | 224-203 | |
| ZNF574 | Forward Primer | ACATTGAGCACCGCTATGTCT | 21 | 61.5 | 32-52 | 186 |
| | Reverse Primer | CCTGCACAAGGGTCTGATAGA | 21 | 60.9 | 217-197 | |
